# Incomplete Kawasaki Disease in a Six-Month-Old Child: A Complex Journey to Recovery

**DOI:** 10.7759/cureus.79778

**Published:** 2025-02-27

**Authors:** Shahd Abouelenen, Nitin Verma, Prashanth Srihari Bhat

**Affiliations:** 1 College of Medicine, Gulf Medical University, Ajman, ARE; 2 Pediatrics, King’s College Hospital London, Dubai, ARE; 3 Neonatology, King’s College Hospital London, Dubai, ARE

**Keywords:** coronary aneurysm, croup, incomplete kawasaki disease, intravenous immunoglobulin, maculopapular erythematous rash

## Abstract

Kawasaki disease (KD) is an acute, self-limited vasculitis that predominantly affects children under five years and can lead to coronary artery aneurysms. Incomplete KD involves an incomplete clinical picture supported by echocardiographic and laboratory findings. In this case, a six-month-old male child presented with a two-day fever, maculopapular erythematous rash, nonpurulent conjunctivitis, throat congestion, mild cough, intermittent loose stools, and was initially diagnosed with a viral illness treated symptomatically. Three days later, he re-presented with unresolved symptoms. Laboratory findings showed significantly elevated inflammatory markers, leukocytosis, and thrombocytosis. Chest X-ray was normal, and IgM measles test, respiratory viral panel, and mycoplasma serology were negative. The fever was managed with antipyretics and intravenous antibiotics, and antibiotic eye drops were given for the conjunctivitis. He improved clinically and repeat investigations showed improvement, following which he was discharged on oral antibiotics and antibiotic eye drops. Three days later (day 10 of illness), he presented with high spiking fever for 48 hours, bilateral nonpurulent conjunctivitis, and exudative tonsillitis. After excluding viral or bacterial etiologies, the patient was further evaluated for KD. Echocardiogram showed dilated coronary arteries, including an evolving aneurysm in the left anterior descending artery, confirming incomplete KD. He was treated with intravenous immunoglobulin and aspirin, leading to the resolution of his symptoms within 24 hours. Follow-up echocardiogram showed improvement and he was discharged on aspirin with scheduled cardiac monitoring. This case highlights the diagnostic challenges of incomplete KD, its potential association with underlying infections, and the effectiveness of timely treatment.

## Introduction

Kawasaki disease (KD) is an acute, self-limited vasculitis of unknown etiology affecting medium-sized vessels and can lead to coronary artery aneurysms, primarily in children under five years [1]. Despite being self-limited, it is important to begin therapy with intravenous immunoglobulin within 10 days of fever onset to reduce the risk of coronary complications [1]. KD is diagnosed clinically and classified as complete or incomplete [2]. The diagnostic criteria for complete KD include fever for five or more days and at least four principal features, namely, oropharyngeal mucositis (e.g., strawberry tongue, erythema, cracked lips), bilateral nonexudative conjunctivitis, maculopapular erythematous rash, extremity changes (e.g., edema, erythema, or periungual desquamation), and cervical lymphadenopathy [2,3]. Incomplete KD is diagnosed when fewer than four features are present, with supportive echocardiographic and laboratory findings such as elevated inflammatory markers (erythrocyte sedimentation rate: ≥40 mm/hour and C-reactive protein: ≥3.0 mg/dL), anemia, or thrombocytosis (platelets: ≥450,000/mm^3^ one week after fever onset) [2,3]. KD can also be classified by phase as acute (up to day 10), subacute (day 10 to week 6.5), and convalescent (weeks 6.5 to 9) [2].

## Case presentation

A six-month-old male child presented to the emergency department (ED) with a spiking fever of up to 38.2°C and a red maculopapular rash since the previous day. The rash started behind the ears and cheeks, gradually spreading to the whole body. He had a mild cough for two weeks before the rash, intermittent loose stools, no vomiting, and normal urine output. Oral intake was good. No significant past medical or surgical history, or known allergies. He was up to date on immunizations.

**Figure 1:**
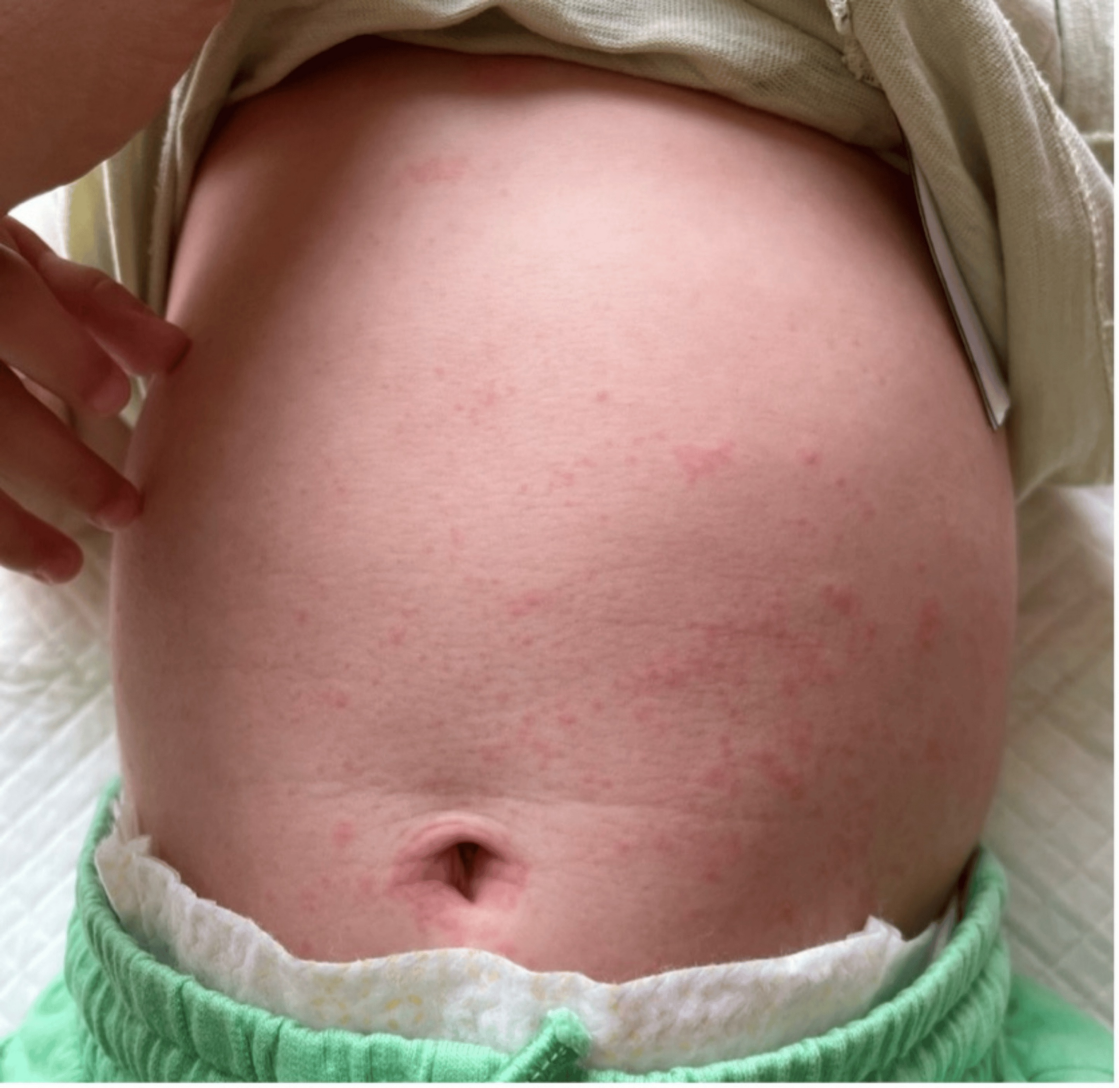
Red maculopapular erythematous rash on the trunk.

**Figure 2:**
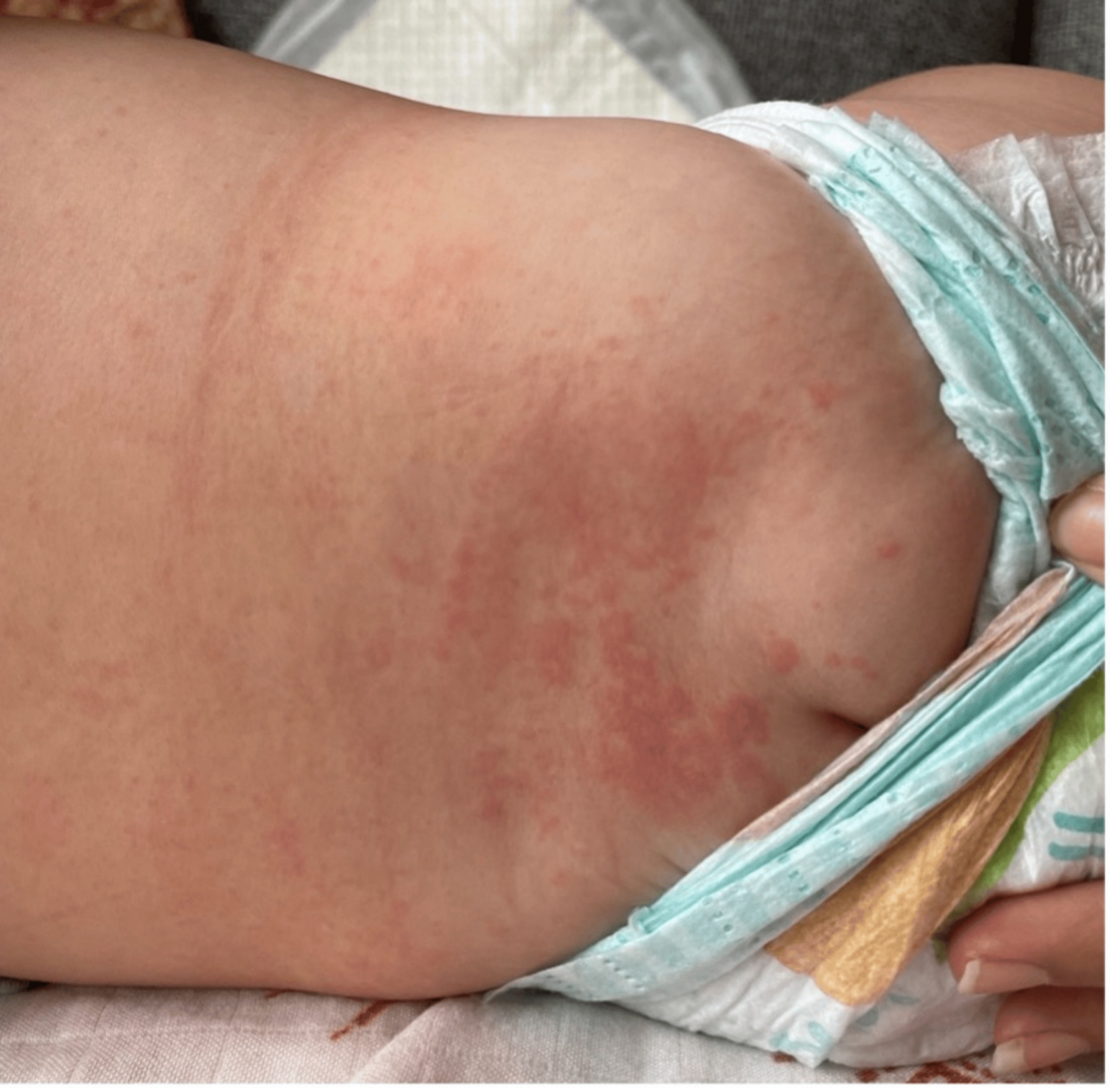
Red maculopapular erythematous rash on the back.

**Figure 3:**
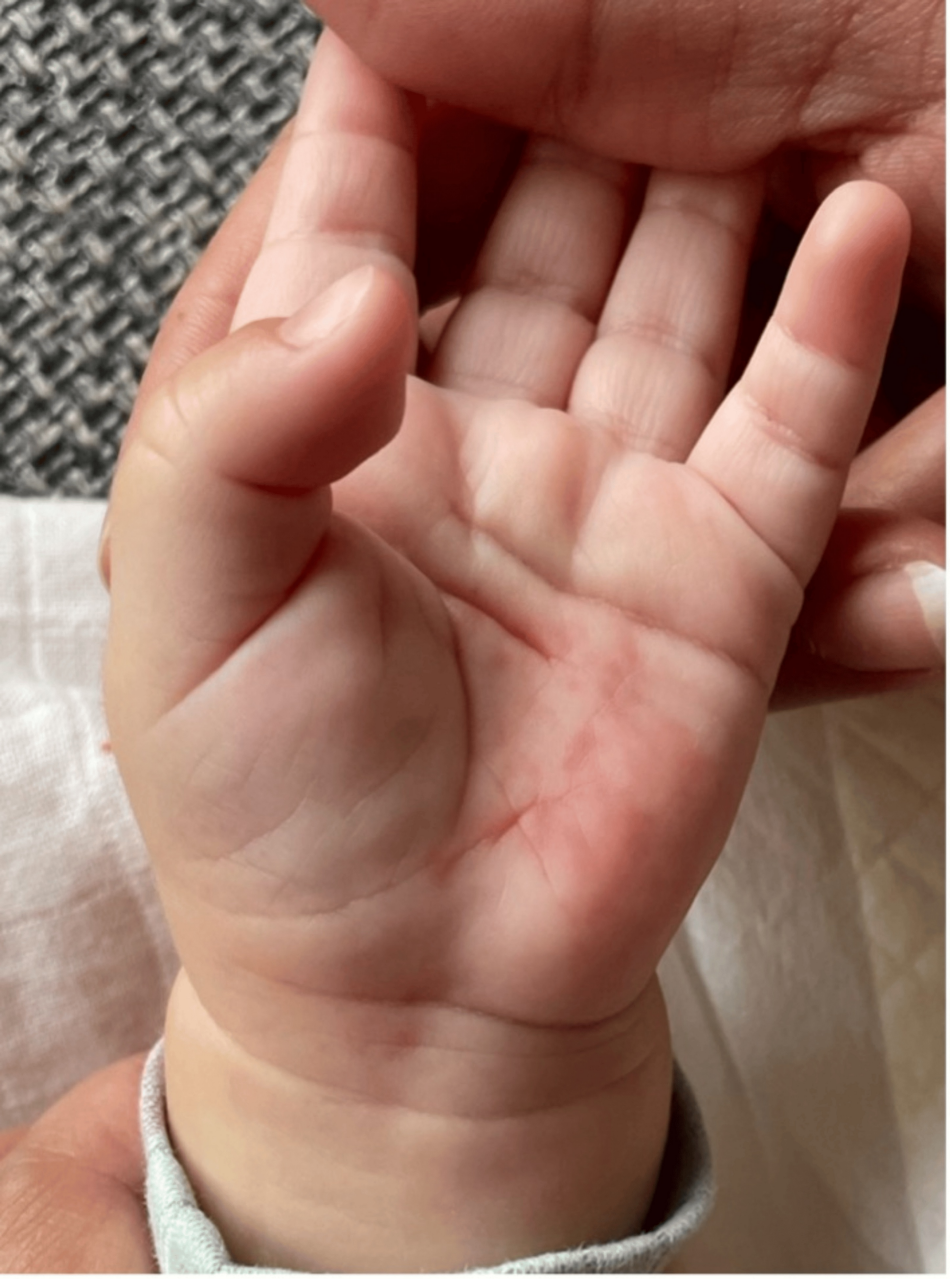
Red maculopapular erythematous rash on the palm.

Upon physical examination, he was alert but miserable, well-nourished, well-hydrated, had nonpurulent conjunctivitis, but no acute respiratory distress. The skin showed a red blanching maculopapular erythematous rash (Figures 1, 2) all over the body, including the soles and palms (Figure 3). The throat was congested. The rest of the systemic examination, including development, was normal. The initial diagnosis was a likely viral illness and was sent home on symptomatic management and safety net advice.

Three days later, he re-presented to the ED with persistent high fever spikes (>39°C), rash similar to the earlier presentation, nonpurulent conjunctivitis, and a cough. Diarrhea had settled down. Fever management included intravenous paracetamol and ibuprofen, two-thirds maintenance intravenous fluids, and intravenous ceftriaxone. Tobramycin two drops every six hours was given for the conjunctivitis.

The results of the investigations showed an elevated white blood cell (WBC) count (21.2 × 10³/μL) with neutrophils at 9.98 × 10³/μL, high platelet count (651 × 10³/μL), and elevated C-reactive protein (CRP) level (95.4 mg/L). The erythrocyte sedimentation rate (ESR) was 44 mm/hour. The alanine aminotransferase (ALT) was elevated (85 IU/L). The bicarbonate level was low (17 mmol/L). Renal function tests were normal. Blood culture was negative. The measles immunoglobulin M test, respiratory viral panel, and mycoplasma serology were all negative. The chest X-ray was normal.

However, the patient developed a croupy cough with a mild stridor on day two of admission which resolved with one dose of oral dexamethasone 0.15 mg/kg and nebulized epinephrine 0.4 µg/kg. The following morning, respiratory examination was normal, CRP decreased to 30.1 mg/L, oral intake improved, and the intravenous fluids were tapered down. His rashes started to fade and became apyrexial after two doses of intravenous ceftriaxone. He was discharged in a stable condition after three doses of intravenous ceftriaxone on oral amoxicillin/clavulanic acid for four days, tobramycin eye drops, and follow-up was arranged.

Three days later, he presented to the ED with spiking fever for 48 hours, bilateral nonpurulent conjunctivitis, and enlarged tonsils with white follicles. There was no swelling or peeling of the extremities, strawberry tongue, or lymphadenopathy. Investigations showed elevated CRP (132 mg/L), ESR (49 mm/hour), WBC count (24.9 × 10³/μL), ALT (33 IU/L), and thrombocytosis (893 × 10³/μL). The respiratory viral panel, antistreptolysin O (ASO) test, and repeat blood culture were negative. An echocardiogram was requested at this point to rule out incomplete KD.

**Figure 4:**
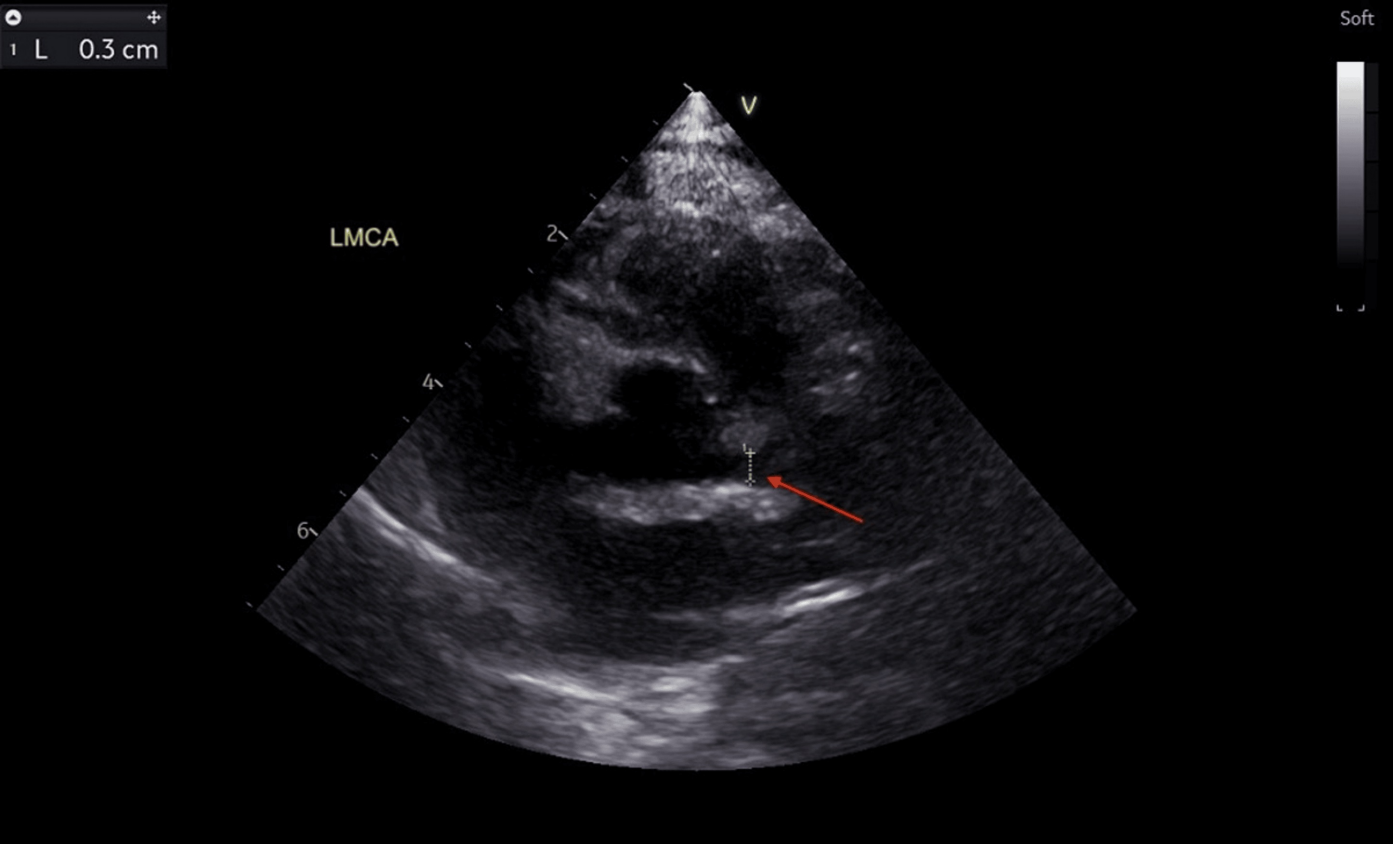
Echocardiogram showing a dilated left main coronary artery. The red arrow points at the dilated left main coronary artery measuring 3.0 mm (Z score: +3.64).

**Figure 5:**
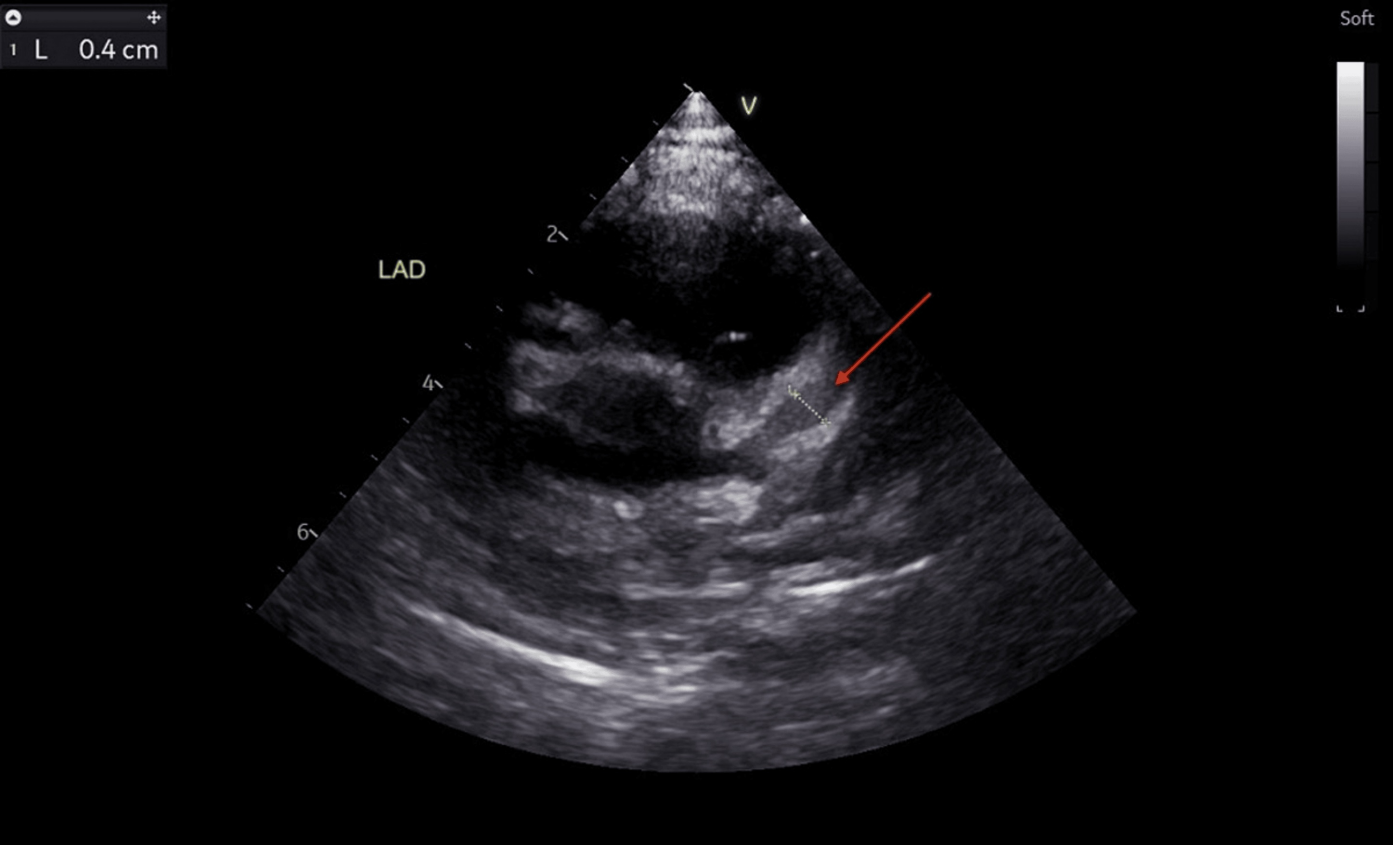
Echocardiogram showing a dilated left anterior descending artery. The red arrow points at the dilated left anterior descending artery measuring 4.0 mm (Z score: +6.72).

**Figure 6:**
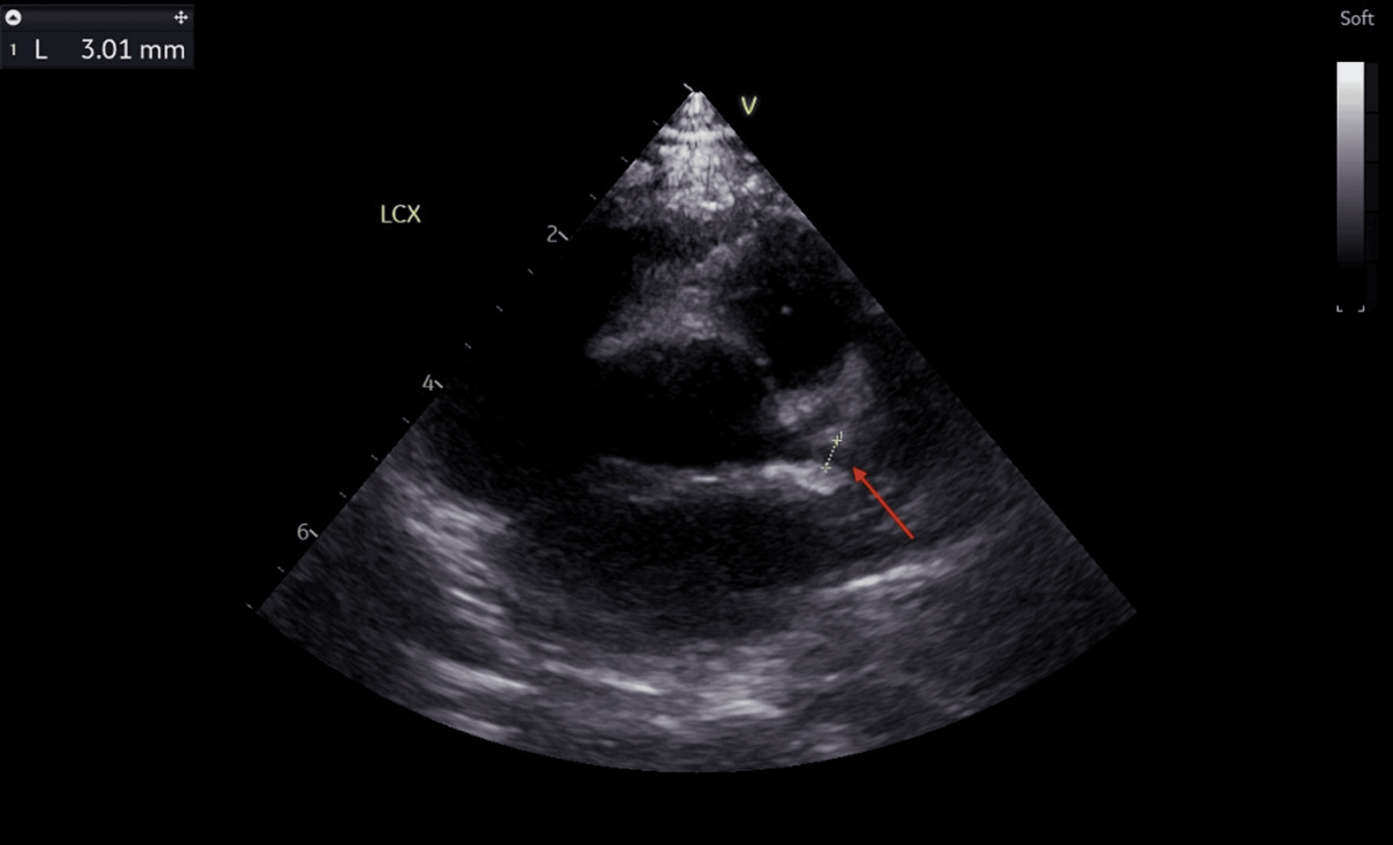
Echocardiogram showing a dilated left circumflex artery. The red arrow points at the dilated left circumflex artery measuring 3.0 mm (Z score: +5.11).

**Figure 7:**
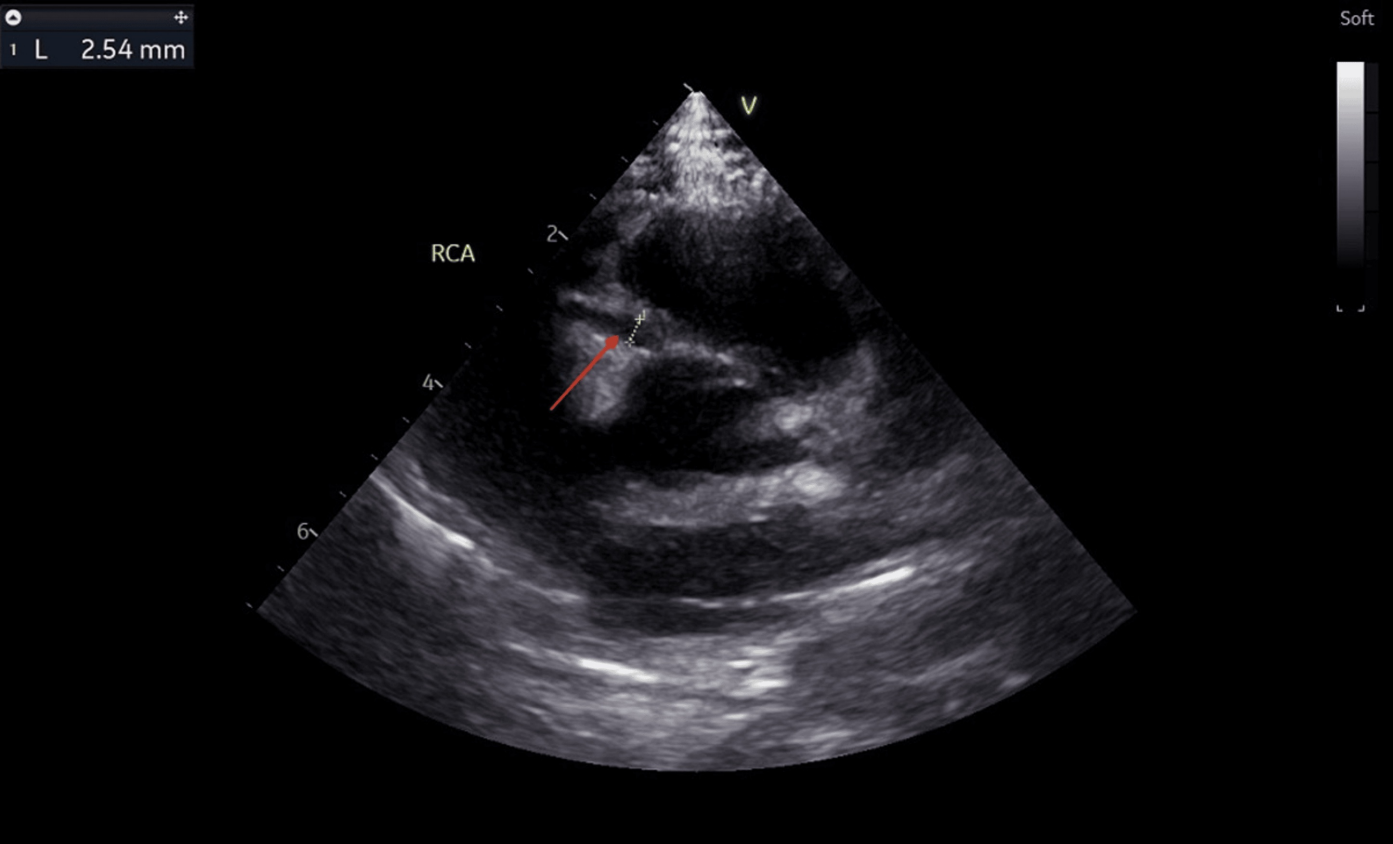
Echocardiogram showing a dilated right coronary artery. The red arrow points at the dilated right coronary artery measuring 2.5 mm (Z score: +2.83).

The findings on the echocardiogram showed dilated coronary arteries, with an evolving aneurysm in the left anterior descending artery (LAD). The left main coronary artery (LMCA) measured 3.0 mm (Z score: +3.64) (Figure 4), LAD appeared dilated with an area of cystic dilatation measuring 4.0 mm (Z score: +6.72) (Figure 5) and the left circumflex artery (LCX) measured 3.0 mm (Z score: +5.11) (Figure 6). The proximal right coronary artery (RCA) measured 2.5 mm (Z score: +2.83) (Figure 7). There was good biventricular systolic and diastolic function, no valvular regurgitation, and no pericardial effusion.

According to the latest American Heart Association guidelines, the patient was treated with a single-dose intravenous immunoglobulin (IVIG) 2 g/kg and oral aspirin 30 mg/kg/day in four divided doses. Esomeprazole was added for gastric protection. He became apyrexial within 24 hours of IVIG, and his rash and conjunctivitis resolved.

**Figure 8:**
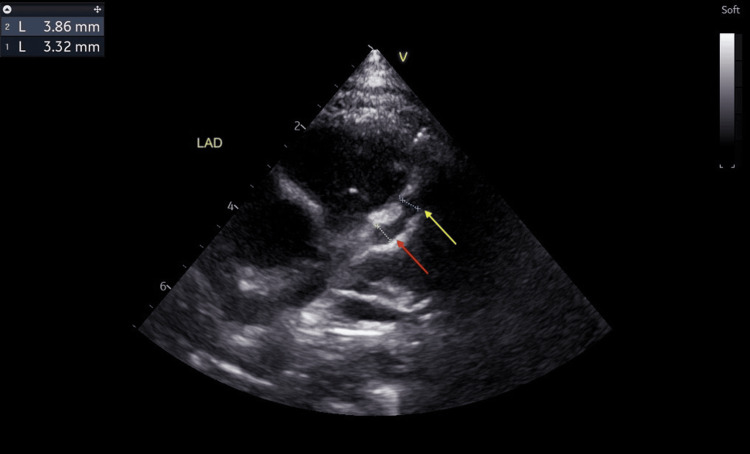
Echocardiogram showing two small fusiform dilatations in the left anterior descending artery. The yellow arrow points at a dilatation measuring 3.32 mm (Z score: +6.16), and the red arrow points at another dilatation measuring 3.86 mm (Z score: +7.84).

**Figure 9:**
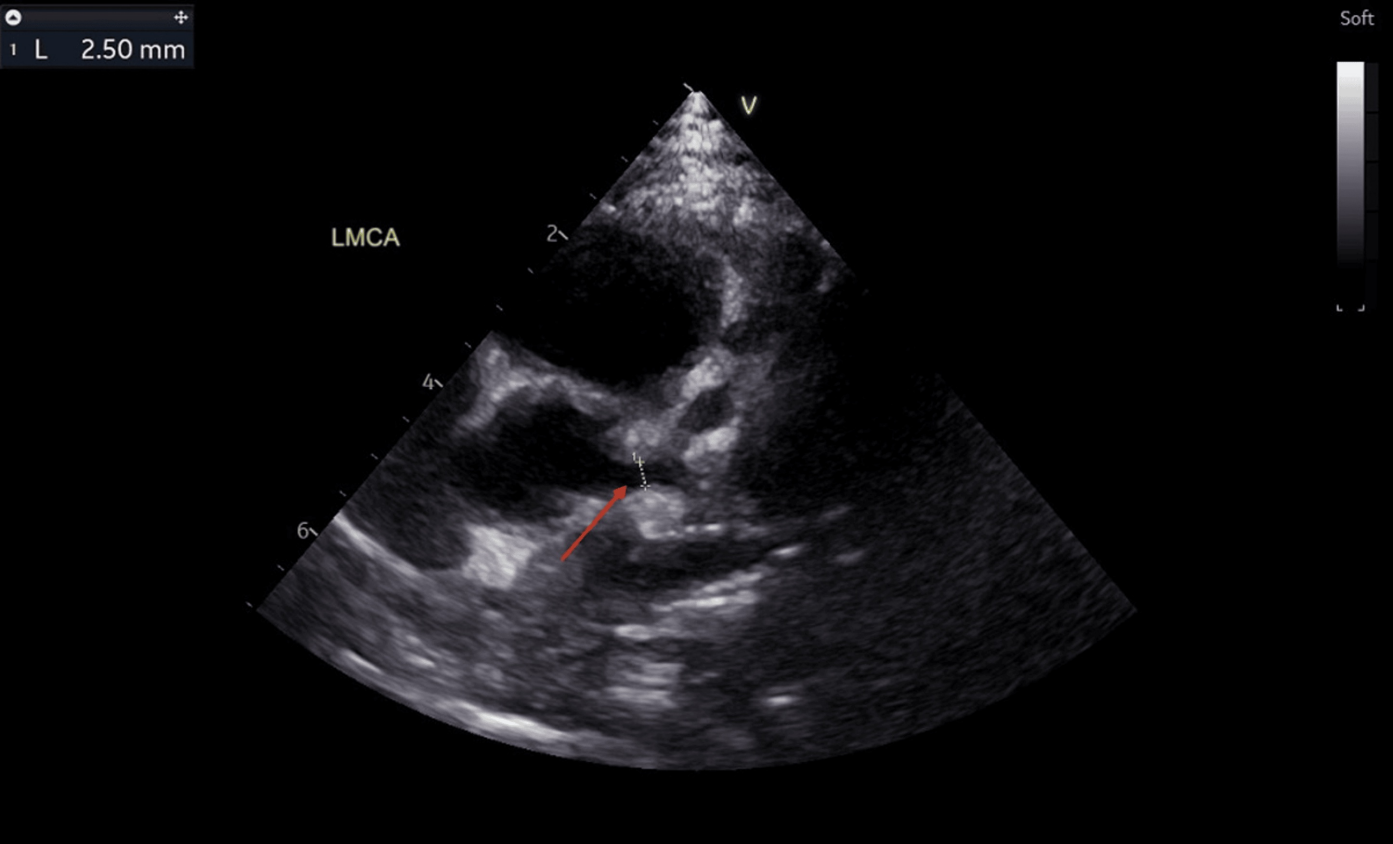
Echocardiogram showing reduction in the left main coronary artery dilatation. The red arrow points at the dilated left main coronary artery measuring 2.50 mm (Z score: +2.20).

**Figure 10:**
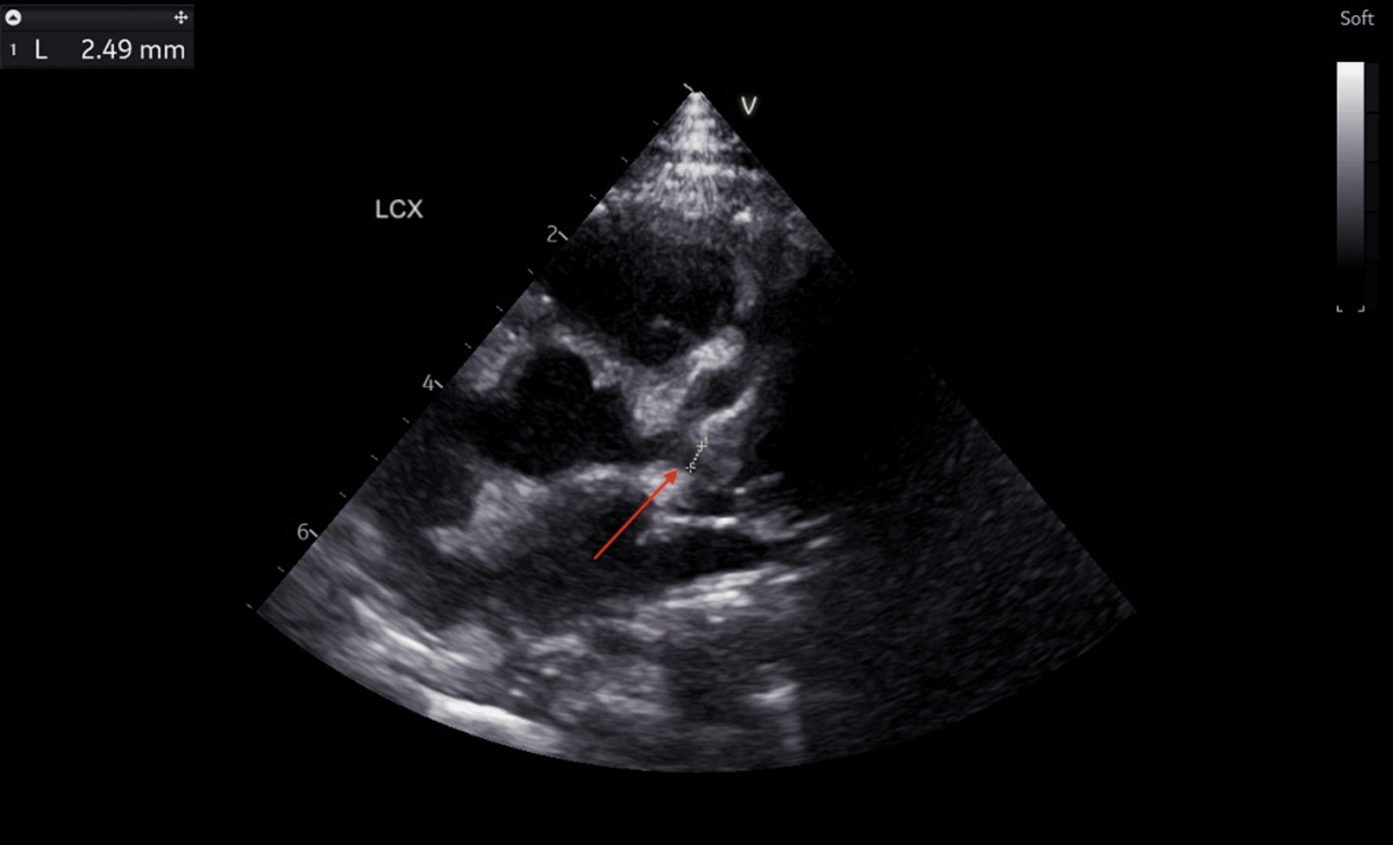
Echocardiogram showing reduction in the left circumflex artery dilatation. The red arrow points at the dilated left circumflex artery measuring 2.49 mm (Z score: +3.60).

**Figure 11:**
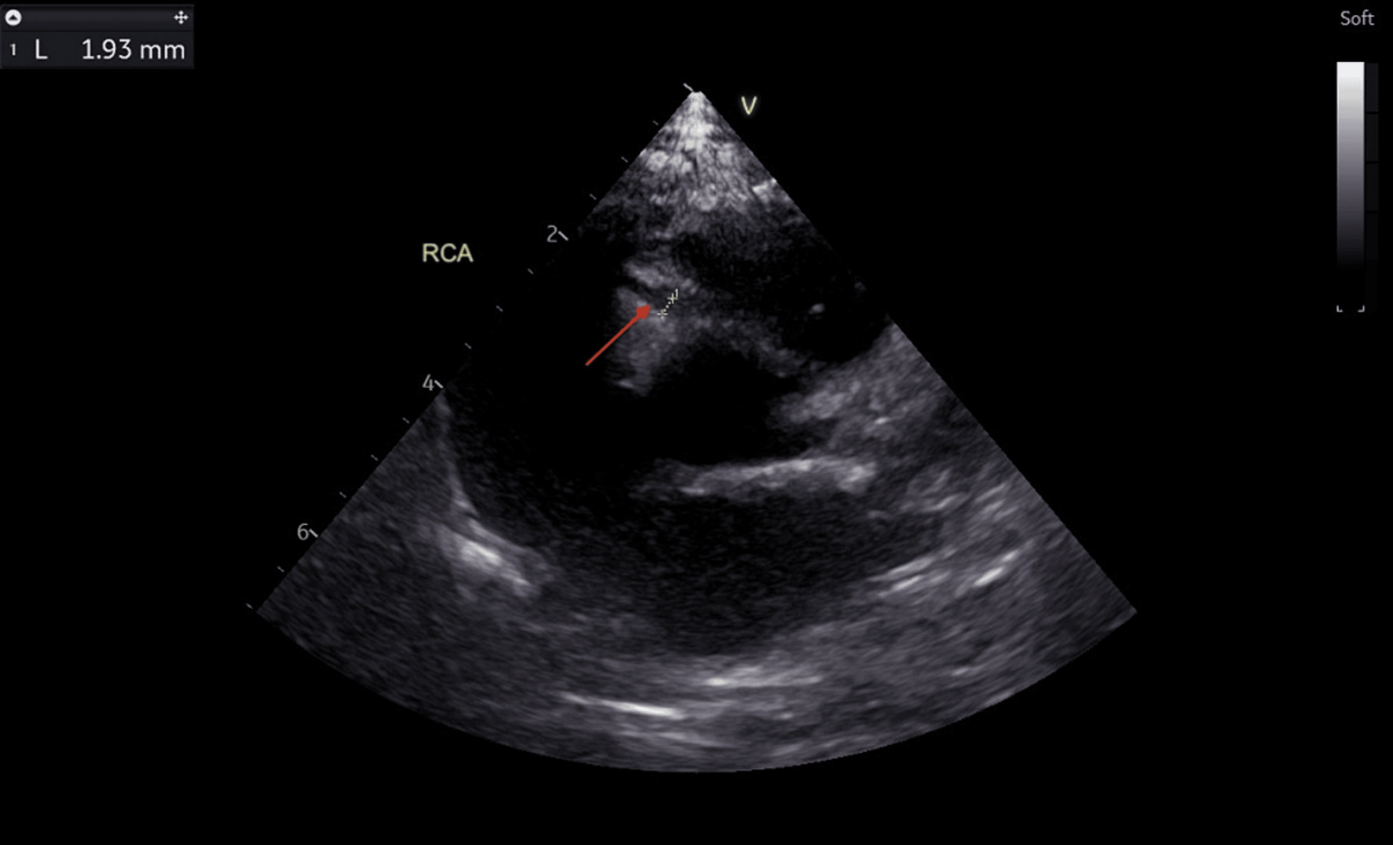
Echocardiogram showing reduction in the right coronary artery dilatation. The red arrow points at the dilated right coronary artery measuring 1.93 mm (Z score: +1.18).

He was discharged after being apyrexial for 48 hours. The CRP had dropped to 20.3 mg/L, ESR to 25 mm/hour, and repeat blood culture was negative. No antibiotic was given during this admission. A repeat echocardiogram at discharge showed two small fusiform dilatations in the LAD measuring 3.86 mm (Z score: +7.84) and 3.32 mm (Z score: +6.16) (Figure 8). However, there was a reduction in the left coronary artery dilatations: LMCA (2.50 mm, Z score: +2.20) (Figure 9), LCX (2.49 mm, Z score: +3.60) (Figure 10). RCA measured 1.93 mm (Z score: +1.18) (Figure 11). Biventricular systolic and diastolic function were good, with no valvular regurgitation and no pericardial effusion.

The plan was to continue oral aspirin for two weeks (30 mg/kg in four divided doses), followed by 5 mg/kg once daily for the next six weeks. Echocardiograms were scheduled at two and six weeks post-discharge and long-term cardiac follow-up arrangements were discussed to monitor for resolution or progression of coronary abnormalities. The details of the patient’s test results are summarized in Table 1.

**Table 1:** Patient test results. ED: emergency department; WBC: white blood cell; CRP: C-reactive protein; ESR: erythrocyte sedimentation rate; ALT: alanine aminotransferase; IgM: immunoglobulin M; ASO: antistreptolysin O; LMCA: left main coronary artery; LAD: left anterior descending artery; LCX: left circumflex artery; RCA: right coronary artery

Investigation	Second ED visit (day five of illness)	Second ED visit (day seven of illness)	Third ED visit (day 10 of illness)	At discharge (day 13 of illness)	Reference range
WBC (×10³/μL)	21.2	-	24.9	-	5.2–14.5
Neutrophils (×10³/μL)	9.98	-	11.88	-	1–4
Platelets (×10³/μL)	651	-	893	-	191–523
CRP (mg/L)	95.4	30.1	132	20.3	0–4.9
ESR (mm/hour)	44	-	49	25	2–34
ALT (IU/L)	85	-	33	-	0–29
Bicarbonate (mmol/L)	17	-	23	-	15–25
Blood culture	Negative	-	Negative	-	Negative
Measles IgM	Negative	-	-	-	Negative
Respiratory viral panel	Negative	-	Negative	-	Negative
Mycoplasma serology	Negative	-	-	-	Negative
ASO	-	-	Negative	-	Negative
Echocardiogram	-	-	LMCA: 3.0 mm (Z score: +3.64)	LMCA: 2.50 mm (Z score: +2.20)	Z score: -2 to +2
			LAD dilatation: evolving 4.0 mm (Z score: +6.72)	LAD dilatation: 3.86 mm (Z score: +7.84) and 3.32 mm (Z score: +6.16)	Z score -2 to + 2
			LCX: 3.0 mm (Z score: +5.11)	LCX: 2.49 mm (Z score: +3.60)	Z score: -2 to + 2
			RCA: 2.5 mm (Z score: +2.83)	RCA: 1.93 mm (Z score: +1.18)	Z score: -2 to + 2

## Discussion

The incidence of KD significantly differs across regions, with North America, Europe, and Australia reporting rates of 5-22 per 100,000 children under five, which have stabilized in the past decade [4]. In contrast, North-Eastern Asian countries, particularly Japan, Korea, and Taiwan, have an incidence over 10 times higher and continue to rise according to data from the last two decades [5]. This regional disparity has encouraged extensive genetic research, identifying susceptibility genes, including single-nucleotide polymorphisms in *ITPKC*, *CASP3*, *FCGR2A*, *BLK*, *ORAI*, and *CD40*, through genome-wide association and linkage analyses across various ethnic populations [5]. These genetic variations have been linked not only to the etiology and prognosis of KD but also to the risk of developing coronary artery aneurysms [5]. On the other hand, data from the Middle East is limited to case reports and small single-center studies, which indicate that KD is not as uncommon as previously assumed, and increased awareness and diagnosis will help understand the true incidence.

The diagnosis of incomplete KD can be challenging, particularly in younger infants who may not present with the classical symptoms. In this case, the patient initially presented with spiking fever, bilateral nonpurulent conjunctivitis, maculopapular erythematous rash, and throat congestion but did not meet the complete diagnostic criteria for KD at the time of initial evaluation. However, the persistence of fever, progressive laboratory abnormalities, including markedly elevated CRP (132 mg/L), ESR (49 mm/hour), thrombocytosis (platelets: 893 × 10³/μL), and the development of coronary artery dilatation on echocardiography confirmed the diagnosis of incomplete KD.

This case provides a valuable learning opportunity, highlighting the diagnostic challenges of incomplete KD. The patient’s initial presentation mimicked a viral infection, and the development of a croupy cough and mild stridor was an unusual feature rarely observed or reported in KD. This suggests the possibility of airway inflammation as a rare association or complication of KD due to extravascular tissue inflammation. Recent large-scale epidemiological studies have revealed significant associations between certain viral infections and KD [6-9].

KD has been linked to respiratory syncytial virus (RSV), human rhinovirus (hRV), rotavirus, and norovirus in Korea [6], as well as RSV, influenza A and B, and human metapneumovirus in Chile [7]. Moreover, studies in Korea and the United States have found associations between KD and RSV, hRV, and varicella [8,9]. A recent Japanese study published in October 2024 reported that seasonal viruses and bacteria contribute to 30-50% of KD cases [10]. However, the exact mechanisms underlying these associations remain unclear. Our case report encourages further research into this emerging and understudied area.

The patient’s transient improvement with antibiotics misled to the suspicion of a bacterial etiology, which is unusual in KD and has not been previously reported. This false resolution contributed to delayed KD-specific treatment with IVIG and aspirin, which are essential in reducing the risk of coronary artery aneurysms. It is important to investigate and further research the mechanism behind this antibiotic response, as it represents an interesting event in KD management, given that previous case reports have consistently shown that the fever associated with KD does not respond to antibiotics [11-13]. Additionally, the timely IVIG and aspirin administration led to a favorable outcome despite initial diagnostic challenges and significant coronary involvement. The patient became afebrile within 24 hours, with echocardiography showing a reduction in coronary dilatations, highlighting the effectiveness of prompt KD-specific treatment.

## Conclusions

This case report highlights the challenges of diagnosing incomplete KD, especially when symptoms mimic viral infections. Recent research suggests a potential association between seasonal viruses and bacteria and the development of KD. The development of croupy cough and mild stridor was a rare manifestation, further emphasizing the uniqueness of this case. Moreover, transient improvement with antibiotics misdirected to the suspicion of a bacterial etiology, which is another unusual finding and encourages research into the mechanism behind this response. Timely administration of IVIG and aspirin resulted in rapid clinical improvement and regression of coronary abnormalities. This underscores the significance of early diagnosis, regular monitoring, and prompt treatment to prevent complications and improve outcomes in incomplete KD.
